# Photocrosslinkable Kidney Decellularized Extracellular Matrix‐Based Bioink for 3D Bioprinting

**DOI:** 10.1002/adhm.202501616

**Published:** 2025-07-16

**Authors:** Jaemyung Shin, Nima Tabatabaei Rezaei, Subin Choi, Zhangkang Li, Deok‐Ho Kim, Keekyoung Kim

**Affiliations:** ^1^ Department of Biomedical Engineering Schulich School of Engineering University of Calgary Calgary Alberta T2N 1N4 Canada; ^2^ Department of Mechanical and Manufacturing Engineering Schulich School of Engineering University of Calgary Calgary Alberta T2N 1N4 Canada; ^3^ Basic Medical Research Center Medical School of Nantong University Co‐Innovation Center of Neuroregeneration Nantong Jiangsu 226001 China; ^4^ Department of Biomedical Engineering Johns Hopkins University Baltimore Maryland 21218 USA

**Keywords:** 3D bioprinting, decellularized extracellular matrix, kidney tissue engineering, photocrosslinkable bioink

## Abstract

Three‐dimensional bioprinting has emerged as a promising strategy in tissue engineering, enabling the fabrication of biomimetic tissue constructs for regenerative medicine, disease modeling, and drug screening. A key challenge in this field is the development of organ‐specific bioinks capable of recapitulating native microenvironments to support cell viability, proliferation, and tissue‐specific maturation. In this study, a novel photocrosslinkable bioink derived from methacrylated decellularized porcine kidney extracellular matrix (KdMA) is reported. The decellularization process effectively removed cellular components while preserving key extracellular matrix constituents. The resulting KdMA bioink exhibited favorable rheological properties, including tunable stiffness and rapid photocuring kinetics, making it compatible with both digital light processing‐based stereolithography and extrusion‐based bioprinting platforms. Encapsulated human embryonic kidney cells maintained high viability and formed multicellular spheroids, demonstrating the bioink's cytocompatibility and structural support. Additionally, the KdMA bioink enabled stable multilayer bioprinting with preserved structural integrity and tunable mechanical properties. These results underscore the utility of KdMA as a kidney‐specific bioink and its promise as a versatile platform for advancing renal tissue engineering and organoid maturation.

## Graphical abstract

A photocrosslinkable bioink is developed from methacrylated decellularized porcine kidney extracellular matrix to create kidney‐specific 3D constructs. This bioink supports high cell viability, promotes multicellular spheroid formation, and enables stable multilayer bioprinting with tunable mechanical properties, providing a versatile platform for fabricating biomimetic renal tissues and advancing organoid development.

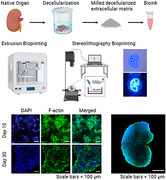

## 1 Introduction

Chronic kidney disease (CKD), which can progress to end‐stage renal disease, is a significant health concern in North America, affecting ≈37 million people in the United States, ≈15% of the adult population,^[^ ^1^, ^2^ ^]^ This condition is a leading cause of morbidity and mortality, contributing to over 100 000 new cases of end‐stage renal disease annually.^[^ ^3^ ^]^ In Canada, CKD affects over 3 million individuals, with a prevalence rate of ≈10% among adults.^[^ ^4^ ^]^ Given the widespread impact and severity of kidney disease, tissue engineering and other bioengineering approaches have emerged as powerful tools for disease modeling and drug screening, offering innovative solutions to address these challenges,^[^ ^5^, ^6^, ^7^ ^]^

Three‐dimensional (3D) bioprinting technology offers significant advantages for biomedical applications, particularly in the development of personalized tissue models, organ prototypes, and patient‐specific implants.^[^ ^8^ ^]^ Its ability to fabricate complex tissue structures with high precision enables better replication of native tissue architecture, which is crucial for applications such as tissue engineering and precision medicine.^[^ ^9^ ^]^ One of the most critical components of bioprinting is the bioink, which must meet essential criteria, including biocompatibility, printability, and biodegradability to ensure successful bioprinting and the functional integration of the bioprinted tissues or organs,^[^ ^10^, ^11^ ^]^

Bioinks are commonly derived from both synthetic and naturally occurring hydrogels. Synthetic biomaterials offer advantages such as customizability, batch‐to‐batch consistency, and scalability, allowing for precise control over properties and large‐scale production.^[^ ^12^ ^]^ However, naturally derived biomaterials, particularly those based on extracellular matrix (ECM), are valued for their biocompatibility and ability to closely replicate the native tissue microenvironment, which is essential for promoting cellular growth and tissue regeneration,^[^ ^13^, ^14^ ^]^ Biological scaffold materials composed of ECM have been shown to promote structural remodeling of various tissues in both preclinical animal studies and human clinical applications,^[^ ^15^, ^16^ ^]^ For instance, Kim et al. reported enhanced vascularization and maturation of human kidney organoids cultured using a kidney decellularized extracellular matrix (dECM) hydrogel.^[^ ^17^ ^]^ Moreover, in the field of tissue engineering, Sobreiro‐Almeida et al. biofabricated a renal 3D microenvironment in vitro using a developed kidney dECM, employing extrusion‐based 3D bioprinting with thermal crosslinking.^[^ ^18^ ^]^

However, dECM, in particular, has poor mechanical properties, making it challenging to utilize for printing applications,^[^ ^19^, ^20^ ^]^ ECM‐based hydrogels are often limited by slow and uncontrollable gelation processes and mechanical properties that fail to replicate physiological conditions. To address these challenges, natural polymers have been functionalized with photoreactive components to enable the rapid formation of hydrogels with tunable gelation properties. Synthetic modifications, including photocross‐linkability, have also been introduced to enhance functionality and mechanical properties. Rezaei et al. developed a methacrylated decellularized extracellular matrix (methacrylated dECM, abbreviated as dMA)‐based bioink from porcine liver and formulated a hybrid bioink by incorporating it with gelatin methacrylate (GelMA).^[^ ^21^ ^]^ The photocross‐linkable dECM‐based bioink preserved the beneficial properties of dECM, as demonstrated by a significant increase in HepG2 cell proliferation and cluster formation, compared to dECM‐free bioinks. However, using highly substituted dMA as a standalone bioink presents significant advantages over hybrid formulations.

With a high degree of substitution (DS) of the methacrylate group, dMA undergoes pure photocross‐linking, eliminating the need for additional polymers like GelMA while maintaining structural integrity. Moreover, the absence of non‐native components reduces the risk of immune responses, making pure dMA an ideal bioink for fabricating biomimetic hepatic tissues. In this study, we developed a kidney‐decellularized extracellular matrix methacrylate (KdMA) bioink for digital light processing (DLP)‐based stereolithography (SLA) and piston‐driven extrusion bioprinting, eliminating the need for supplementary materials. Additionally, we aimed to evaluate whether the KdMA hydrogel, without the use of additional photocross‐linkable hydrogel such as GelMA, could promote tissue formation of human embryonic kidney (HEK) cells at a level comparable to the commonly used, well‐established GelMA bioink.^[^ ^22^ ^]^ While prior studies, such as those by Ali et al.,^[^ ^23^ ^]^ have introduced photocross‐linkable kidney‐derived dECM bioinks, our approach presents key innovations, including the use of an immersion/agitation decellularization method rather than perfusion, detailed characterization of crosslinking kinetics, mechanical tunability down to 0.67 kPa, and transparent reporting of bioprinter specifications and performance. These distinctions address critical gaps in the replicability and functional applicability of kidney‐specific bioinks. These findings contribute to the development of organ‐specific bioinks tailored for high‐precision bioprinting techniques, advancing kidney tissue modeling and biofabrication strategies for regenerative applications.

## 2 Results and Discussion

### 2.1 Development of Kidney dECM and KdMA Bioink

**Figure 1:**
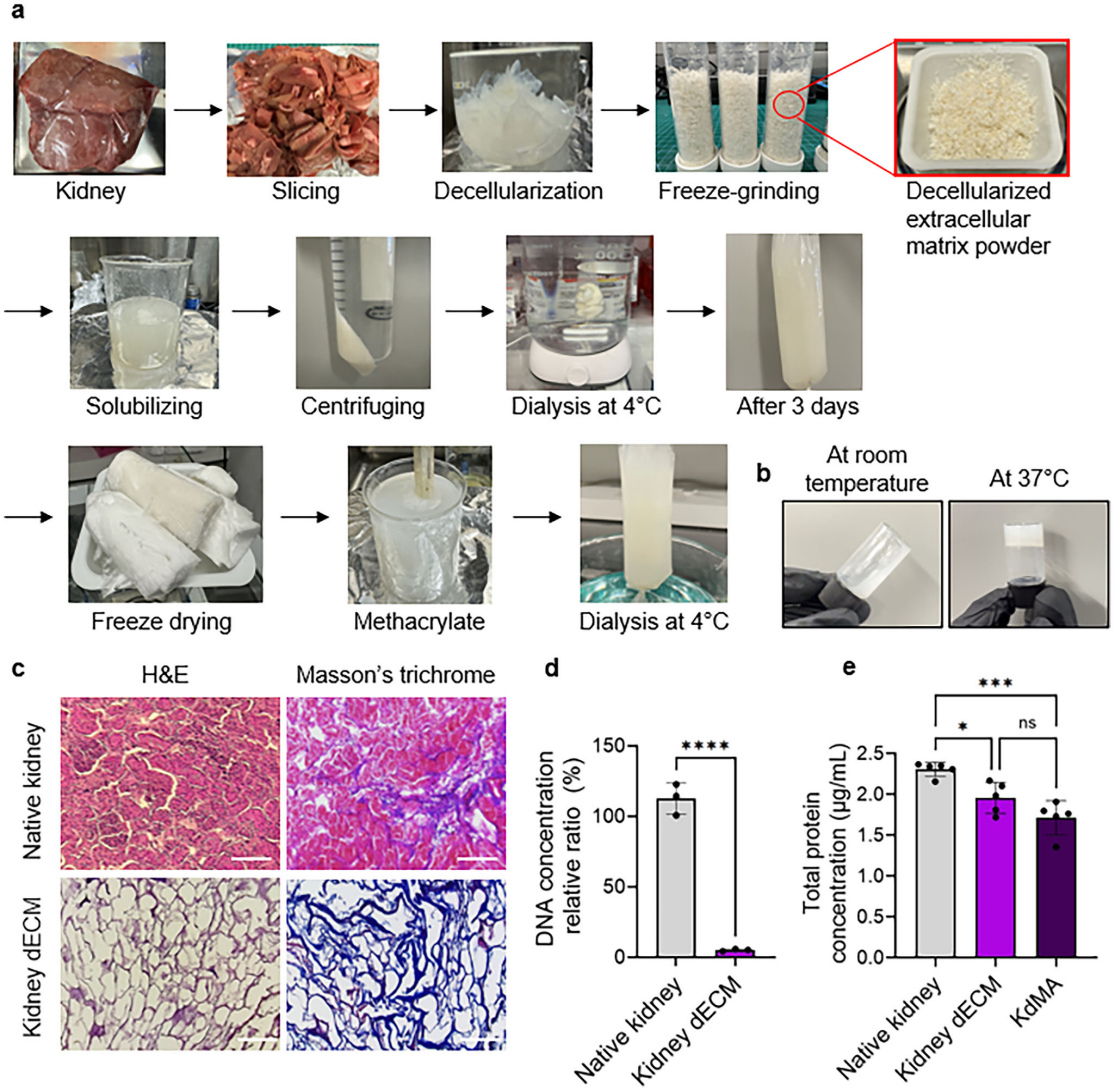
a) A comprehensive overview of the process for making kidney dECM hydrogel followed by synthesizing KdMA from porcine kidney tissue. b) Thermal gelation behavior of kidney dECM hydrogel demonstrating the transition from sol to gel at physiological temperature. c) H&E and Masson's trichrome staining of the native kidney and KdMA, Scale bars = 100 µm. d) Comparison of total DNA content between the native kidney and kidney dECM (*n* = 5; ** p < 0.01). e) Comparative analysis of TPC among the native kidney, kidney dECM, and KdMA. (*n* = 5; * p < 0.05, *** p < 0.001). Error bars represent standard deviation.

To develop a kidney dECM bioink suitable for bioprinting, a decellularization process was performed using a fresh porcine kidney (**Figure** **1**a). The process was optimized to ensure the complete removal of porcine cells while preserving key ECM components, such as fibrous proteins, basement membranes, and proteoglycans. As shown in Figure 1b and Movie S1 (Supporting Information), the kidney dECM exhibited thermocross‐linking behavior, remaining in a liquid state at room temperature but undergoing sol‐gel transition and solidifying at 37 °C. This behavior can be attributed to its collagen content, which enables temperature‐dependent self‐assembly and gelation.

To further investigate its composition, histochemical staining was performed to visualize cellular nuclear material and collagen content. Figure 1c presents representative cross‐sectional images of native kidney tissue stained with hematoxylin and eosin (H&E), revealing well‐defined nephron structures, including glomeruli, and organized cellular arrangements within the kidney cortex, thereby reflecting the intact architecture of the kidney. In contrast, H&E staining of the kidney dECM showed a structure devoid of dark, purple‐stained nuclei, confirming successful cell removal. Additionally, the presence of collagen fiber in the kidney dECM was verified through Masson's trichrome staining, which highlighted collagen fibers in blue (Figure 1c). These results confirm that collagen fibers remain the primary structural component following decellularization.

To quantitatively assess the efficiency of decellularization, deoxyribonucleic acid (DNA) and total protein content (TPC) were analyzed. The results showed that the DNA content of the kidney dECM decreased to less than 5.21% of the native kidney DNA content (Figure 1d). This corresponds to an absolute residual DNA level of 5.21 ng mg^−1^ dry weight, which is well below the commonly accepted thresholds for 50 ng/mg for decellularized biomaterials, as defined by Crapo et al.^[^ ^24^ ^]^ Therefore, the residual DNA content is considered acceptable for translational applications. Although ionic detergents such as sodium dodecyl sulfate (SDS) are highly effective in cell removal, they are more aggressive than non‐ionic treatments and tend to deplete ECM proteins to a greater extent. To evaluate the impact of the washing and functionalization steps on TPC, measurements revealed a statistically significant difference in TPC between native kidney tissue and the final KdMA product. However, no statistically significant difference was observed in TPC between kidney dECM and KdMA, indicating that the functionalization process did not induce additional protein loss (Figure 1e).

### 2.2 Degree of Methacrylation

**Figure 2:**
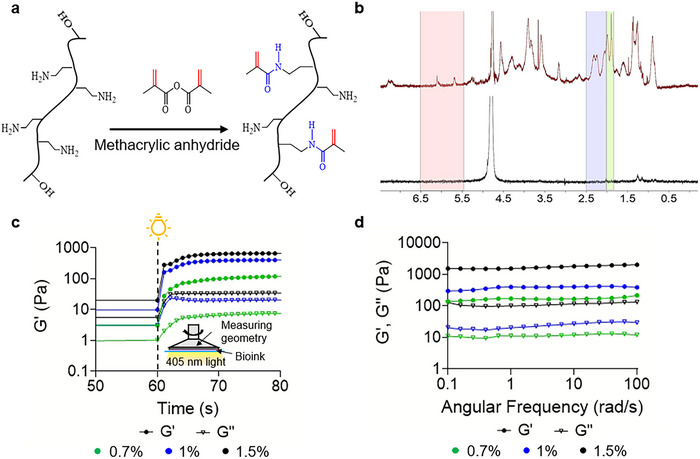
a) Mechanism of methacrylate group substitution on KdMA backbone molecules. b) Characterization of the DS of methacrylate group in KdMA, as demonstrated by ^1^H NMR spectra of KdMA and kidney dECM samples. c) Photocuring kinetics of KdMA hydrogels, represented by storage modulus (G’) and loss modulus (G’’) with 0.5% LAP. d) Angular frequency response of KdMA bioink synthesized with 0.5% LAP.

The abundant collagen proteins within the synthesized kidney dECM were utilized to modify the amine (‐NH_2_) functional groups in the primary collagen framework, allowing for the substitution with methacrylate groups. The methacrylation reaction of kidney dECM using methacrylic anhydride to synthesize KdMA is shown in **Figure** **2**a. This reaction follows a mechanism similar to GelMA synthesis, where both GelMA and KdMA undergo methacrylation through comparable chemical processes. This illustration is included to help understanding, as the methacrylation processes for both GelMA and KdMA follow similar chemical reaction mechanisms. The methacrylation of collagen requires dissolution; therefore, pepsin was used to enzymatically digest the matrix, partially hydrolyzing non‐fibrillar collagen regions to facilitate solubilization while preserving its triple‐helical structure, which is essential for maintaining bioactivity. This enzymatic digestion also ensured a homogeneous solution, enhancing reactivity with methacrylic anhydride for efficient methacrylation.

To assess the DS of methacrylate groups, proton nuclear magnetic resonance (^1^H NMR) analysis was performed (Figure 2b). KdMA exhibited new peaks at 5.29 and 5.72 ppm, corresponding to the methacrylate end groups, and at 1.97 ppm, corresponding to the methacrylamide end groups of the macromolecules. A peak near 4.79 ppm was observed in both dECM and KdMA spectra, which corresponds to the residual semi‐deuterated water signal from the D_2_O solvent, and it is not indicative of any structural modification. Additionally, a peak near 4.0 ppm was observed specifically in the KdMA spectrum but not in the native dECM. This signal is attributed to the methylene (CH₂) protons adjacent to the introduced methacryloyl groups, which are commonly seen as triplets or multiplets in this region following methacrylation of primary amine (‐NH₂) or hydroxyl (‐OH) groups. Such a peak is absent in the unmodified dECM, which primarily contains native protein structures without these methacrylated side chains. For internal reference, the glycine *α*‐CH proton signal at 3.5–3.8 ppm was selected, as it appeared consistently at the same chemical shift in both spectra. Peak integration relative to the internal reference revealed a DS of ≈61.5%.^[^ ^11^, ^12^ ^]^

To further enhance the degree of methacrylation, an additional grinding step using a cryomilling machine before the solubilization process is expected to improve methacrylation efficiency. Cryomilling reduces particle size and increases surface area, facilitating greater exposure of reactive sites and promoting more effective methacrylation. This hypothesis is based on preliminary visual observations from ongoing work, which confirm that cryomilled dECM tissue is noticeably finer than non‐cryomilled samples, suggesting enhanced solubilization and functionalization potential,^[^ ^25^, ^26^ ^]^ This process not only enhances the activation of methacrylate groups but also improves material transparency by refining it into fine powders. Consequently, it significantly reduces the stirring time required to achieve a homogeneous KdMA solution, which currently takes three days, thereby enabling the production of a ready‐to‐use biomaterial with improved efficiency.

### 2.3 Photocross‐linking Characterization

The photorheological properties of the bioink were investigated using photorheological testing, which monitored changes in the storage modulus (G') and loss modulus (G“”) under blue light irradiation, as shown in Figure 2c. Changes in G' and G“” during the photopolymerization process provide valuable insights into the material's behavior during crosslinking. The point at which the G' and G“” curves intersect is typically considered the gel point of the material. Interestingly, for the KdMA, no clear gel point was observed within the light curing time. This suggests that G' exceeded G“” even before light exposure, likely due to the presence of collagen fibers forming a partial network through entanglement before full light‐induced gelation. This behavior was more pronounced in higher‐viscosity inks, indicating that polymer concentration influences pre‐crosslinking interactions.

Gelation time, defined as the period from the onset of light exposure to the initial increase in G', varied among the samples. In our experiment, light irradiation began at 60 s, and all KdMA formulations (0.7%, 1%, and 1.5%) exhibited a rapid increase in G’. The storage modulus reached a stable plateau by ≈70 s, indicating that full crosslinking occurred within 10 s of light exposure. This observation is directly supported by the rheological curves in Figure 2c, which clearly demonstrate the rapid photo‐induced gelation behavior of the bioinks. This rapid crosslinking process is crucial for maintaining the structural integrity of the printed construct. However, the lowest concentration bioink exhibited a more gradual increase in storage modulus upon light exposure compared to the other two formulations, indicating that the 0.7% KdMA bioink is the softest and undergoes a slower crosslinking process.

A key consideration in SLA printing is the ability to cleanly separate the printed structure from the uncured bioink, which is essential for achieving high printing resolution. Optimal SLA printing requires high rigidity in the printed structure and low viscosity in the uncured bioink. ∆G' is quantified as the logarithmic difference between the G’ values of the cross‐linked and uncross‐linked states, highlighting the rheological disparity between the printed structure and the uncured hydrogel. Throughout the photocuring process, as the bioink undergoes increased crosslinking, the ∆G’ value rises until crosslinking is complete, at which point it stabilizes. However, variations in mechanical properties, as previously discussed, were reflected in the different final G' and G“” values after cross‐linking, which increased with rising KdMA concentration, thereby elevating ∆G’ as well. In conclusion, higher KdMA concentrations resulted in shorter crosslinking times due to the greater availability of methacryloyl groups and reduced free radical diffusion pathways, leading to the formation of a stiffer network. This property enhances printability and mechanical stability, making higher concentrations more suitable for applications requiring precise structural fidelity.

The rheological stability of the photocross‐linked bioink was further assessed using a frequency sweep test, where G’ and G’’ were measured as a function of angular frequency (rad/s) (Figure 2d). As the values remained stable without significant fluctuations for up to 5 min, the graph is shown only up to 100 s for improved data visualization. All bioink formulations exhibited a consistent, linear trend without significant fluctuations, indicating that the bioink maintained a stable crosslinked network after photocuring. The absence of frequency dependence in G’ and G’’ suggests that the bioink achieved full crosslinking, forming a structurally stable hydrogel. This result confirms that the photocross‐linked bioink exhibits gel‐like behavior with minimal viscoelastic variation across different angular frequencies, which is crucial for maintaining mechanical integrity after bioprinting.

### 2.4 Physical Property Characterization

#### 2.4.1 Mechanical Properties

**Figure 3:**
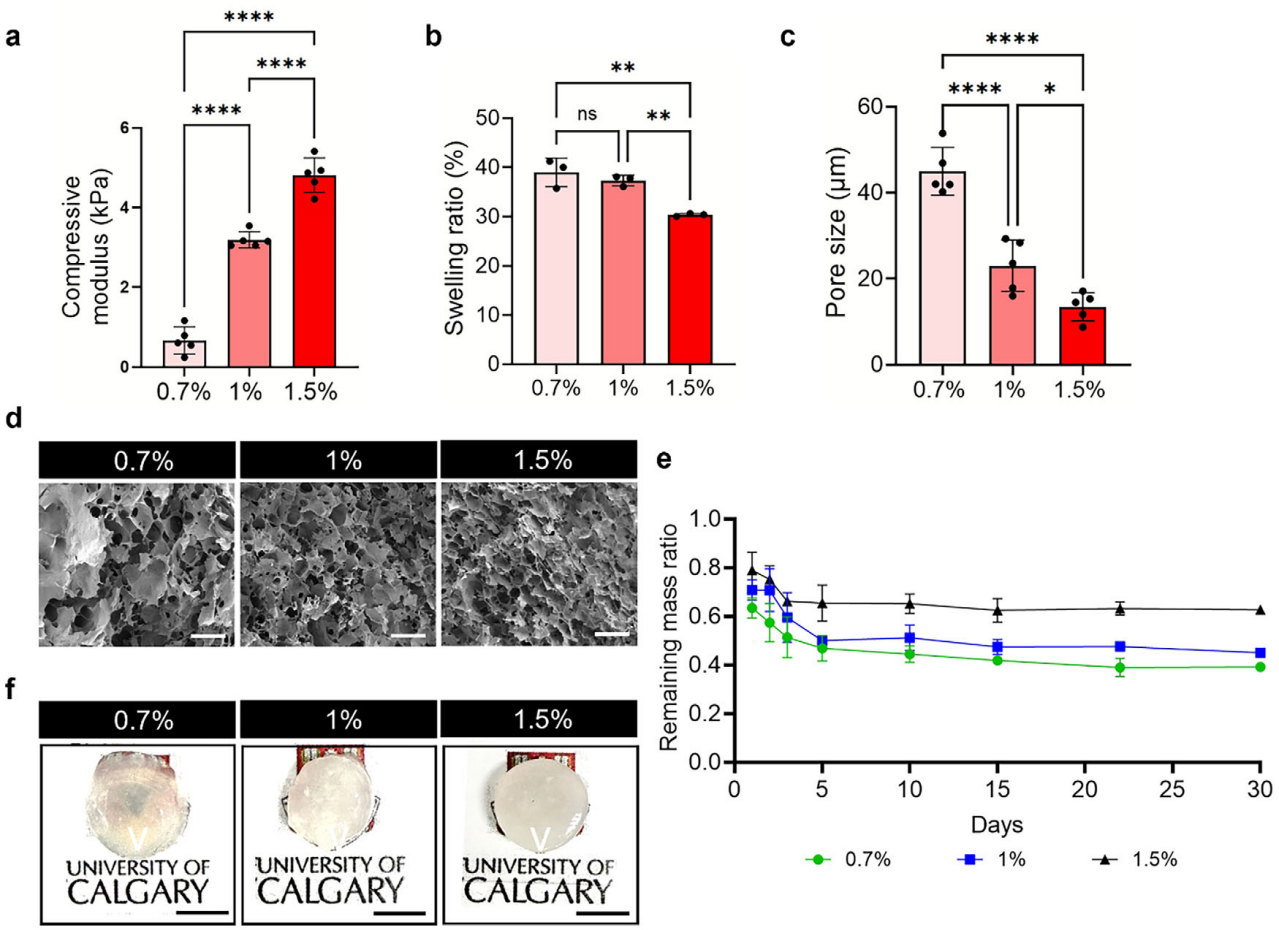
a) Compressive modulus of KdMA with varying compositions (*n* = 5). b) Mass swelling ratio of KdMA with varying compositions (*n* = 5). c) Pore size analysis of the 3D microstructure of the KdMA (*n* = 5). d) SEM images showing the porous structure, Scale bars = 200 µm. e) Degradation analysis of the KdMA immersed in sterilized PBS (*n* = 3). f) Transparency of crosslinked, disk‐shaped hydrogels with varying KdMA compositions, Scale bars = 5 mm. Error bars represent standard deviation.

To investigate the relationship between different KdMA concentrations and compressive modulus, the compressive modulus of KdMA at varying concentrations was measured. Mixing for less than three days resulted in the presence of fine fibers that hindered crosslinking, while stirring for more than a week reduced crosslinking efficiency (Figure S1, Supporting Information). As the concentration increased from 0.7% to 1.5%, a significant increase in compressive modulus was observed (**Figure** **3**a). The measured compressive moduli for 0.7%, 1%, and 1.5% KdMA were 0.67, 3.19, and 4.81 kPa, respectively. These results demonstrate that the compressive modulus can be increased up to sevenfold compared to 0.7% KdMA. This tunable mechanical property makes the developed KdMA bioink a promising biomaterial for various kidney tissue modeling applications.

Material stiffness plays a critical role in replicating the native kidney tissue environment.^[^ ^27^ ^]^ Notably, nephron progenitor cells thrive in a soft environment with a stiffness range of 0.1–3 kPa, which supports kidney organoid formation.^[^ ^27^ ^]^ Therefore, the development of a soft bioink was a key objective of this study. Various studies have explored the mechanical properties of healthy kidney tissue at different developmental stages, as well as in diseased or cancerous kidneys.^[^ ^28^ ^]^ Ruiter et al. investigated human induced pluripotent stem cell (iPSC)‐derived kidney organoids, designed to mimic human kidney organogenesis, under environments ranging from 0.1 to 20 kPa.^[^ ^27^ ^]^ Organoids cultured in a 20 kPa hydrogel exhibited a significant reduction in interstitial and loop of Henle cells compared to those grown in 0.1 and 3 kPa environments. Additionally, organoids cultured in the 20 kPa hydrogel showed a marked decrease in lumen structures, along with the absence of key kidney cell types. These findings indicate that the stiffness and stress relaxation properties of the surrounding environment directly influence kidney organoid development, with soft hydrogels enhancing lumen structure maturation. The tunable mechanical properties of the KdMA hydrogel developed in this study, with adjustable stiffness ranging from 0.67 to 4.81 kPa, highlight its potential to promote kidney organoid maturation.

Furthermore, engineered ECM can be tailored to mimic pathological conditions, such as fibrosis, providing a platform for functional transplant development and advancing disease modeling applications. As the kidney is classified as a soft organ with a shear modulus of ≈4.5 kPa, kidney organoids naturally prefer a soft matrix that closely mimics the native ECM,^[^ ^29^, ^30^, ^31^ ^]^ Nerger e*t al*. encapsulated stem cell‐derived kidney organoids on day 7 of differentiation in Matrigel, Collagen Type I, and alginate hydrogel, conducting a comparative analysis. Their results demonstrated that organoids encapsulated in alginate hydrogel exhibited stable differentiation without significant cell migration, with all major nephron segments remaining present even after 21 days of differentiation.^[^ ^32^ ^]^ They also demonstrated that kidney organoids exhibited improved growth and development in a soft and viscoelastic hydrogel, highlighting the importance of ECM mechanics in organoid maturation.

Building upon these findings, our next study aims to encapsulate iPSC‐derived kidney organoids within a KdMA bioink to further enhance maturation and angiogenesis, creating a more physiologically relevant microenvironment. The bioink developed in this study addresses the poor printability of dECM, significantly enhancing its printability. It also offers a versatile microenvironment by allowing precise control over its mechanical properties through simple adjustments to light exposure and material concentration. This adaptability will be highly advantageous for the bioprinting of kidney organoids.^[^ ^33^ ^]^ Furthermore, by encapsulating renal cells within the developed bioink and culturing them for extended periods, a more physiologically relevant drug screening platform can be established compared to traditional 2D culture methods. This highlights the bioink's versatility and its potential for a wide range of biomedical applications.

#### 2.4.2 Swelling Behavior

The swelling ratio of hydrogels is a critical parameter in tissue engineering, as it directly influences surface properties, solute diffusion, and overall bioink performance.^[^ ^34^ ^]^ Swelling behavior is particularly important in bioprinted scaffolds, as it affects cell migration, nutrient transport, and mechanical stability. Assessing the swelling ratio of crosslinked hydrogel samples provides insights into the degree of crosslinking, water retention capacity, and potential structural stability in biological environments.^[^ ^35^ ^]^ As shown in Figure 3b, the swelling ratio decreased with increasing KdMA concentration, indicating that higher methacrylation levels led to greater crosslinking density, thereby reducing water absorption capacity. The 0.7% KdMA hydrogel exhibited the highest swelling ratio at 38.99%, whereas higher KdMA concentrations resulted in significantly lower water retention. For instance, the swelling ratio of 1.5% KdMA was measured at 30.35%, ≈1.28 times lower than that of 0.7% KdMA. Interestingly, the difference in swelling ratio between 0.7% and 1% KdMA was not statistically significant, suggesting that the water retention capacity reaches a plateau beyond a certain threshold of methacrylation and crosslinking density. This trend may indicate that, at lower KdMA concentrations, the bioink retains sufficient hydration to support cell viability and metabolic activity, while higher crosslinking densities result in a more rigid and less hydrophilic network.

These findings are consistent with previous studies on methacrylated ECM‐based hydrogels, where increased crosslinking led to a reduction in swelling due to a denser polymeric network.^[^ ^21^ ^]^ This property is crucial for applications requiring stable mechanical performance, as excessive swelling can lead to scaffold deformation, compromised structural integrity, and unwanted variations in mechanical properties over time. Conversely, a moderate swelling ratio is beneficial for encapsulated cells, as it facilitates the exchange of oxygen, nutrients, and metabolic waste.

The tunable swelling behavior of KdMA hydrogels offers potential advantages for different applications in renal tissue engineering. For example, lower concentrations (0.7–1% KdMA) may be ideal for kidney organoid culture, where a hydrated, cell‐friendly microenvironment is essential, while higher concentrations (1.5% KdMA) may be better suited for applications requiring enhanced mechanical stability, such as bioprinted scaffolds for long‐term implantation. These results suggest that precisely modulating the degree of methacrylation and bioink concentration can provide an effective strategy for optimizing the mechanical and biological performance of KdMA‐based hydrogels, depending on the specific application. Future studies will focus on evaluating the impact of swelling behavior on long‐term cell viability and differentiation, particularly in the context of kidney organoid development and functional tissue formation.

#### 2.4.3 Microstructure Analysis

To further evaluate the morphology, porosity, and surface characteristics of the hydrogel, scanning electron microscopy (SEM) was performed.^[^ ^36^ ^]^ SEM analysis revealed that increasing KdMA concentration led to a decrease in pore size, which correlated with enhanced mechanical stiffness and a reduced swelling ratio (Figure 3c). As shown in Figure 3d, the 1.5% KdMA hydrogel exhibited a compact microstructure with an average size of 13.47 µm. This dense pore network is consistent with its higher cross‐linking density, which contributes to greater mechanical stiffness and lower water absorption capacity. In contrast, the 0.7% KdMA hydrogel exhibited significantly larger pores, averaging 44.98 µm, which correlated with its lower compressive modulus and higher swelling ratio.

The observed microstructural variations are consistent with previous studies on ECM‐derived hydrogels, where higher polymer concentrations result in a denser network due to increased intermolecular crosslinking.^[^ ^37^ ^]^ A finer pore structure enhances mechanical stability but may also affect cellular infiltration and diffusion of nutrients and oxygen, which are critical for tissue engineering applications. Conversely, larger pore structures, as observed in the 0.7% KdMA hydrogel, may facilitate better cell migration and nutrient transport but at the cost of reduced mechanical integrity. These findings suggest that KdMA bioinks can be tailored for specific applications by modulating concentration‐dependent microstructural properties. For instance, lower concentrations (0.7%) may be advantageous for applications requiring enhanced permeability and bioactivity, such as kidney organoid culture, while higher concentrations (1.5%) may be preferable for applications demanding structural stability, such as scaffold‐based tissue engineering.

Further investigations will focus on how microstructural variations influence long‐term cell behavior, including cell adhesion, proliferation, and differentiation. Understanding these relationships will be essential for optimizing KdMA‐based bioinks for renal tissue engineering and organoid maturation.

#### 2.4.4 Degradation Kinetics

Biodegradability is a critical characteristic for hydrogel‐based scaffolds in tissue engineering applications, as it determines how long the scaffold can maintain its structural integrity and support cellular functions.^[^ ^38^ ^]^ The developed KdMA hydrogel exhibited a slow degradation rate, maintaining its structure for more than 30 days in phosphate‐buffered saline (PBS) (Figure 3e). Among the tested formulations, the 1.5% KdMA hydrogel exhibited the highest remaining mass ratio over the 30 days. This is likely attributed to the increased degree of cross‐linking resulting from the higher concentration of methacrylate groups in KdMA. A higher crosslink density requires the disruption of a greater number of covalent bonds during degradation, thereby delaying structural collapse.^[^ ^39^ ^]^ Additionally, microstructural evaluations revealed that as KdMA concentration increased, pore size decreased, leading to reduced PBS absorption and a slower degradation rate. This inverse correlation between crosslink density and degradation rate is consistent with previous findings in methacrylated ECM‐derived hydrogels.^[^ ^40^ ^]^

The ability to precisely control the degradation rate of KdMA hydrogels suggests their potential as versatile biomaterials for a range of applications, including in vivo implantation and long‐term in vitro studies.^[^ ^41^ ^]^ For instance, slower‐degrading formulations may be more suitable for applications requiring prolonged structural support, such as scaffold‐based renal tissue engineering, whereas faster‐degrading variants could be optimized for applications where scaffold remodeling and matrix turnover are desired, such as kidney organoid culture or bioink‐assisted tissue repair.

Figure 3f illustrates the transparency of the crosslinked scaffold, revealing that its transparency is lower than expected. While the transparency of the prepolymer solution is particularly critical for SLA‐based printing, post‐crosslinking transparency should also be considered, as it directly impacts subsequent imaging analyses, such as fluorescence staining and confocal microscopy.^[^ ^42^ ^]^ Therefore, future efforts should focus on optimizing cryomilling to enhance bioink transparency, which could improve light penetration during printing and facilitate high‐resolution imaging for tissue characterization.^[^ ^25^, ^43^ ^]^

### 2.5 Biocompatibility Assessment

**Figure 4:**
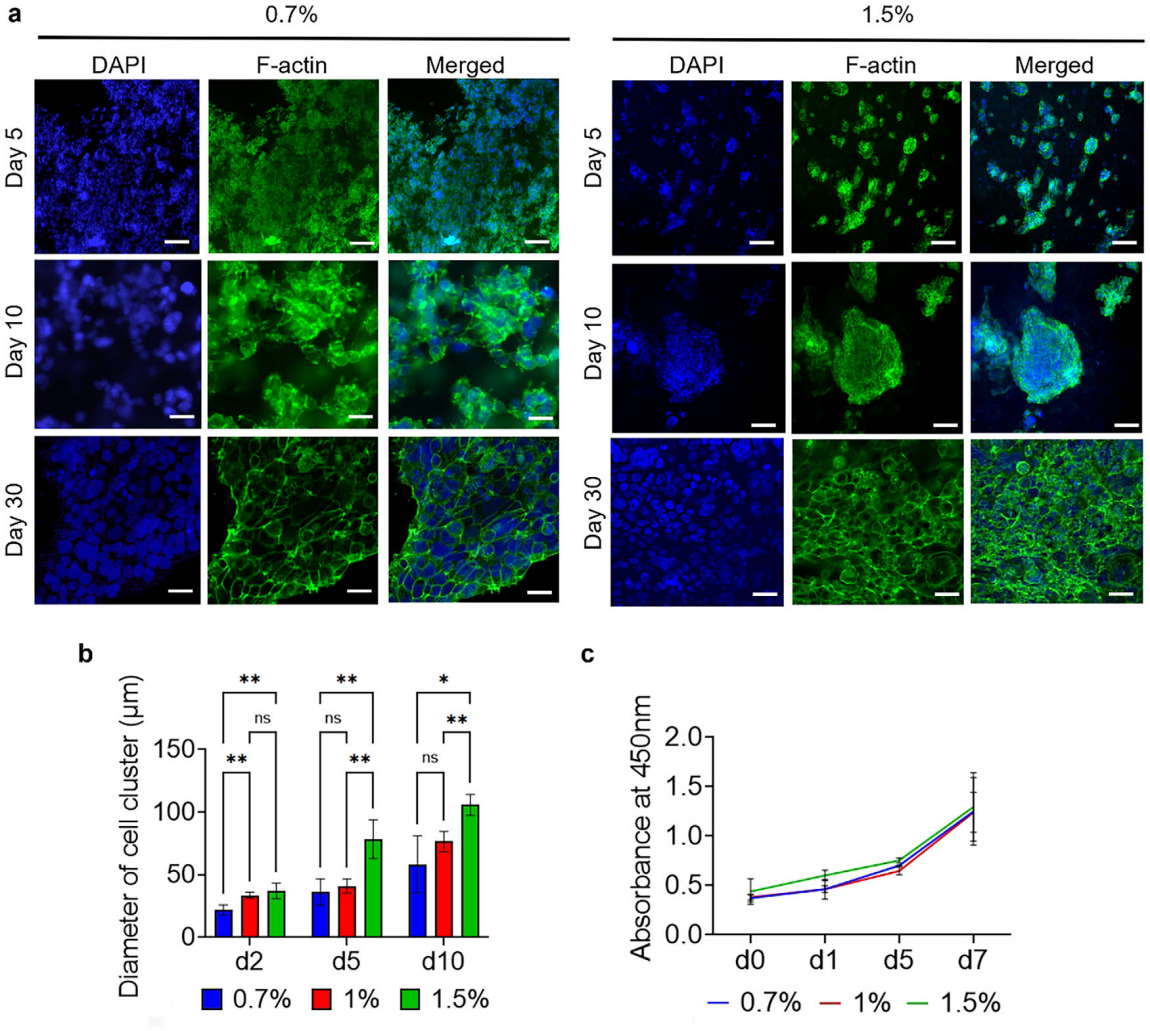
a) Assessment of HEK cell growth, proliferation, and morphology over a 30‐day culture period. Representative fluorescence images showing cell morphology at different time points, with phalloidin‐stained F‐actin (green), DAPI‐stained nuclei (blue), and merged channels, Scale bars = 100 µm. b) Quantification of HEK cell cluster size distribution at various time points during culture (*n* = 5). c) XTT assay evaluating HEK cell metabolic activity and proliferation over 7 days of culture within the 3D hydrogel (*n* = 5). Error bars represent standard deviation.

Cell cytoskeleton and nuclei staining were performed to evaluate the biocompatibility of the developed bioink. The effect of varying bioink concentrations on HEK cell cluster size was also investigated. The bioprinted scaffolds were cultured for one month. Over time, HEK cells progressively developed from individual cells into cohesive clusters and eventually formed multicellular spheroids, consistent with previous studies.^[^ ^44^ ^]^ These spheroids remained viable and stable throughout the study period, exhibiting uniform distribution and proliferation across the entire 3D scaffold up to day 10. In higher concentration bioink samples (1.5% KdMA), cluster size was significantly larger on days 5 and 10 compared to the 0.7% KdMA samples, indicating enhanced cell proliferation (**Figure** **4**a). These clusters exhibited well‐defined cytoskeletons and progressively formed larger multicellular aggregates over time. By day 30, all KdMA formulations demonstrated the presence of proliferating and merging spheroids, suggesting that ECM proteins and growth factors within the dECM scaffold played a significant role in promoting cellular diffusion and proliferation. While these results confirm the cytocompatibility and structural support provided by the KdMA bioink, it is important to note that HEK cells are a transformed embryonic kidney cell line and do not reliably express mature renal markers, such as aquaporin‑1 or megalin. As such, the current study does not evaluate kidney‐specific identity or functionality. These limitations will be addressed in future work using iPSC‐derived nephron progenitor cells and functional assays, including marker expression and proximal tubular function tests.

To further assess cell growth dynamics, the diameter of cell clusters was continuously tracked throughout the culture period. In all bioink formulations, HEK cell cluster size increased over time, with the 1.5% KdMA formulation producing the largest clusters (Figure 4b). To evaluate cell metabolic activity, a 2,3‐bis(2‐methoxy‐4‐nitro‐5‐sulfophenyl)‐2H‐tetrazolium‐5‐carboxanilide (XTT) assay was performed, confirming that the developed bioink effectively supported HEK cell proliferation (Figure 4c). Among the tested conditions, the 1.5% KdMA bioink exhibited the steepest growth rate during the first five days of culture, further demonstrating its capacity to sustain robust cellular activity.

The formation of multicellular spheroids within the bioprinted scaffolds suggests that the developed KdMA bioink provides a favorable microenvironment for HEK cell proliferation and aggregation. The increase in cluster size over time, particularly in higher concentration bioinks (1.5% KdMA), indicates that greater crosslinking density and ECM components contribute to enhanced cellular interactions and stability. The observed cell proliferation and metabolic activity, as confirmed by the XTT assay, highlight the bioink's ability to support sustained cell viability. Similar trends have been reported in dECM‐based hydrogels, where native ECM components facilitate cellular adhesion and growth. These findings suggest that KdMA bioink may serve as a promising platform for engineering complex kidney tissue models, with tunable properties to modulate cellular organization and maturation.

### 2.6 Stereolithography Printing of Cell‐Laden Kidney Microtissues

**Figure 5:**
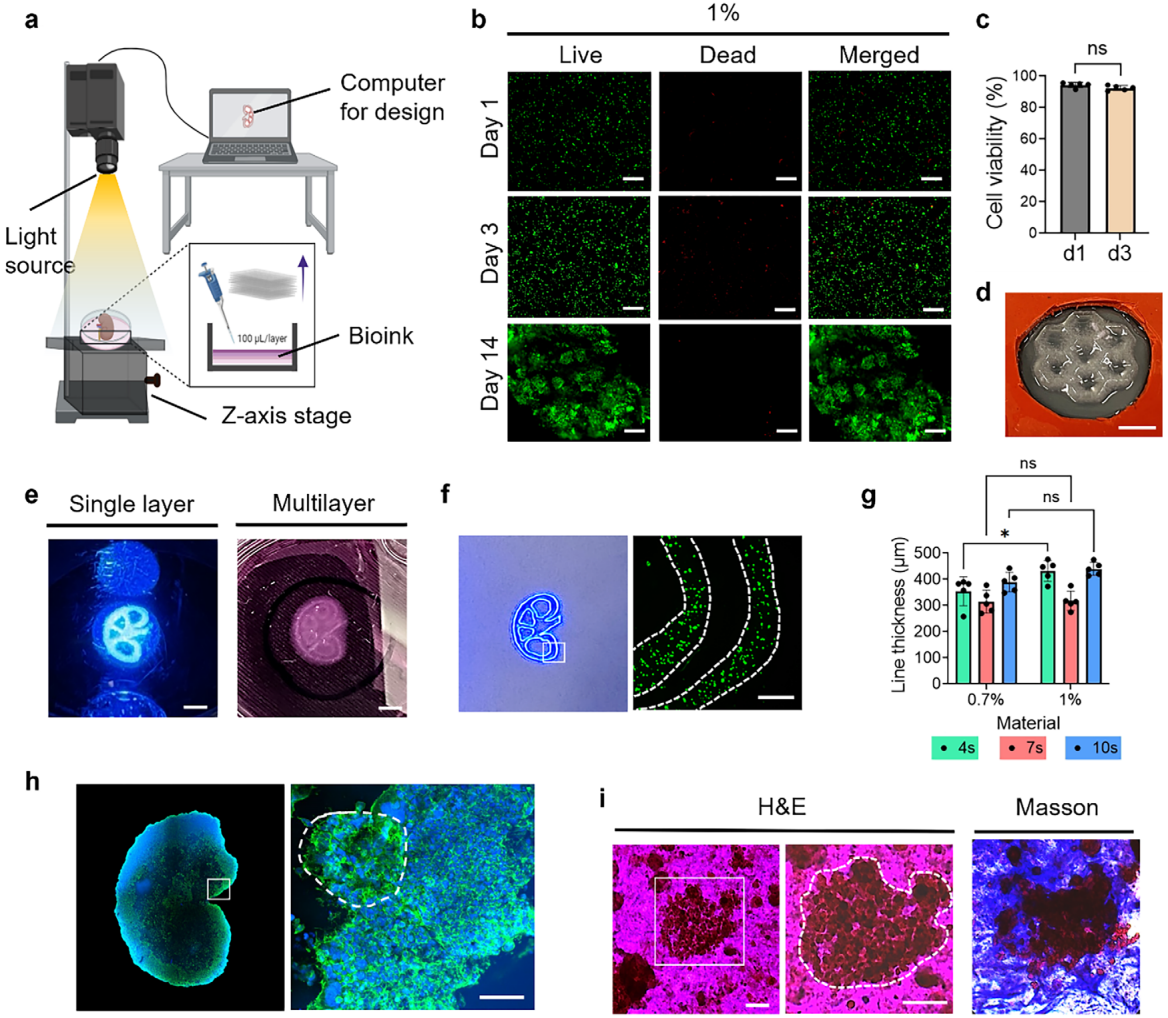
a) DLP‐SLA bioprinting setup equipped with a 405 nm light source and a vertically moving stage, enabling multilayer bioprinting. b) Representative fluorescence images showing live (green) and dead (red) HEK cells encapsulated in hydrogels at various time points over 14 days of culture, Scale bar = 100 µm. c) HEK cell viability (%) within the 3D bioprinted scaffold at day 1 and day 3, (*n* = 5). d) Fabrication of a 3D honeycomb‐shaped structure using 1% KdMA bioink via DLP‐SLA bioprinting, Scale bar = 1 cm. e) The left image shows single‐layer printing after 4‐second crosslinking with 1% KdMA, while the right image presents an intermediate intersection during the printing process, Scale bar = 5 mm. f) Edge‐line printing of the kidney scaffold using 1% KdMA bioink to evaluate line thickness and visualize encapsulated GFP‐tagged 3T3 cells, Scale bars = 50 µm g) Effect of crosslinking time (4, 7, and 10 s) on the printed line thickness (µm) of 0.7% and 1% KdMA bioinks (*n* = 5). h) Confocal imaging of a 3D multilayered mini kidney microtissue fabricated via bioprinting with 1% KdMA bioink, Scale bar = 100 µm. i) Histological assessment of a 0.7% KdMA bioprinted construct following 14 days of HEK cell culture, using H&E and Masson's trichrome staining, Scale bar = 50 µm. Error bars represent standard deviation.

The printability and biocompatibility of the developed bioink were evaluated using a customized DLP‐SLA bioprinter (**Figure** **5**a; Figure S3, Supporting Information). To assess the impact of the printing process on cell viability, HEK cells were encapsulated within the bioink and cultured for 14 days. As shown in Figure 5b, cell viability remained above 90% throughout the culture period, indicating excellent cytocompatibility of both the bioink formulation and the DLP‐SLA bioprinting process. Further viability analysis of bioprinted cell‐laden scaffolds confirmed over 90% viability on both days 1 and 3 post‐printing (Figure 5c). These findings suggest that the photopolymerization process did not exert significant cytotoxic effects and that the bioprinted constructs effectively supported cell survival and proliferation. The high post‐printing viability underscores the suitability of the developed bioink for light‐based bioprinting applications, ensuring that both structural integrity and cellular functionality are maintained.

The structural integrity and multilayer assembly of the bioprinted scaffolds were further examined to assess the fidelity of the DLP‐SLA bioprinting process. Figure 5d illustrates this by showing the bioprinting of a honeycomb‐shaped 3D scaffold using 1% KdMA bioink containing 0.5% Lithium phenyl 2,4,6 trimethyl (LAP), demonstrating the versatility of the printing process (Movie S2, Supporting Information). As illustrated in Figure 5e, the left panel depicts a single‐layer construct, while the right panel captures the bioprinting process at an intermediate stage of multilayer fabrication. As additional layers were stacked, the intersections in the scaffold design became less distinguishable due to increased optical density and structural complexity (Figure 5e). To enhance visualization of the scaffold architecture, the image was captured when approximately half of the total layers had been printed. The printing resolution was further evaluated by selectively printing only the outline of the design using fine lines, as shown in Figure 5f. Additionally, it demonstrates the precise fabrication of two thin lines, ≈100 µm in width, in the lower right section of the kidney‐shaped design, highlighting the capability of the bioink to support high‐resolution printing. To quantitatively assess printing resolution, the line thickness of structures printed with 0.7% and 1% KdMA bioinks was measured under two crosslinking conditions (Figure 5g). At 4 s of light exposure, the printed lines measured 354.28 µm (0.7%) and 432.42 µm (1%), indicating variability due to insufficient cross‐linking. When the crosslinking time was increased to 7 s, line thicknesses improved to 314.52 µm (0.7%) and 317.74 µm (1%), demonstrating enhanced printing fidelity and resolution.

To investigate long‐term cellular organization, the bioprinted scaffolds were cultured for two weeks, followed by confocal microscopy imaging. Phalloidin and 4',6‐diamidino‐2‐phenylindole (DAPI) staining were performed to visualize cell proliferation and structural organization, confirming that the bioprinted constructs facilitated the formation of a 3D mini kidney‐like structure (Figure 5h). These findings demonstrate the potential of the developed bioink for kidney tissue engineering applications, supporting precise bioprinting, cell viability, and tissue formation.

Previous studies, such as the work by Visscher et al. have demonstrated the viability of kidney ECM‐derived bioinks for extrusion‐based bioprinting, showing successful cell proliferation within printed constructs.^[^ ^45^ ^]^ However, our approach introduces several significant advancements and unique characteristics that differentiate it from existing methods. First, we developed a cost‐effective and faster immersion‐based decellularization process compared to the perfusion method. Then, this dMA bioink was used to fabricate a 3D kidney scaffold via the DLP‐SLA bioprinting method. This printing method enabled higher‐resolution printing through layer‐by‐layer cross‐linking of the bioink using 405 nm light, achieving faster printing speeds. Also, we printed the KdMA bioink using the custom‐modified extrusion bioprinter to verify its printability and cellular biocompatibility. Using KdMA, we successfully promoted cellular regeneration in renal tissue bioprinting.^[^ ^17^ ^]^

### 2.7 Histology Assessment

Histological characterization was performed to evaluate cellular morphology and interactions within HEK cells cultured on KdMA scaffolds (Figure 5i). To highlight cellular organization and substrate interaction, H&E staining was applied to 0.7% KdMA scaffolds after 14 days of culture. In the stained samples, HEK cells exhibited well‐defined cytoplasmic structures with distinct nuclei, which appeared vivid blue due to hematoxylin staining, while the cytoplasm was stained bright pink by eosin. This interaction reflects eosin's affinity for cytoplasmic protein components, highlighting intracellular protein organization within the cells.

The ECM plays a crucial role in cellular function and gene expression regulation, directly influencing intracellular protein synthesis.^[^ ^46^ ^]^ The intense cytoplasmic staining observed in HEK cells within KdMA scaffolds suggests active protein synthesis, potentially indicating enhanced cellular maturation. To further investigate the scaffold composition and stability, Masson's trichrome staining was performed to confirm the presence of collagen fibers within the scaffolds supporting HEK cell culture. The 0.7% KdMA samples demonstrated that the intrinsic collagen fibers remained structurally intact and resistant to degradation, even after two weeks of culture, due to covalent crosslinking within the bioink. This finding highlights the long‐term stability of the scaffold, reinforcing the advantages of KdMA bioink for extended kidney cell culture and potential tissue engineering applications.

The histological assessment confirmed that the KdMA bioink provides a supportive microenvironment for HEK cells, as evidenced by well‐preserved cellular morphology and active cytoplasmic staining. The presence of intact collagen fibers, as demonstrated by Masson's trichrome staining, suggests that the scaffold maintains structural stability over extended culture periods, which is critical for long‐term tissue development. These findings indicate that KdMA bioink successfully mimics native ECM properties, promoting cell adhesion, proliferation, and potential maturation. Future studies will focus on evaluating ECM remodeling and long‐term cellular differentiation to further assess its potential for kidney tissue engineering applications.

### 2.8 Rheological Property Analysis for Extrusion‐Based Bioprinting

**Figure 6:**
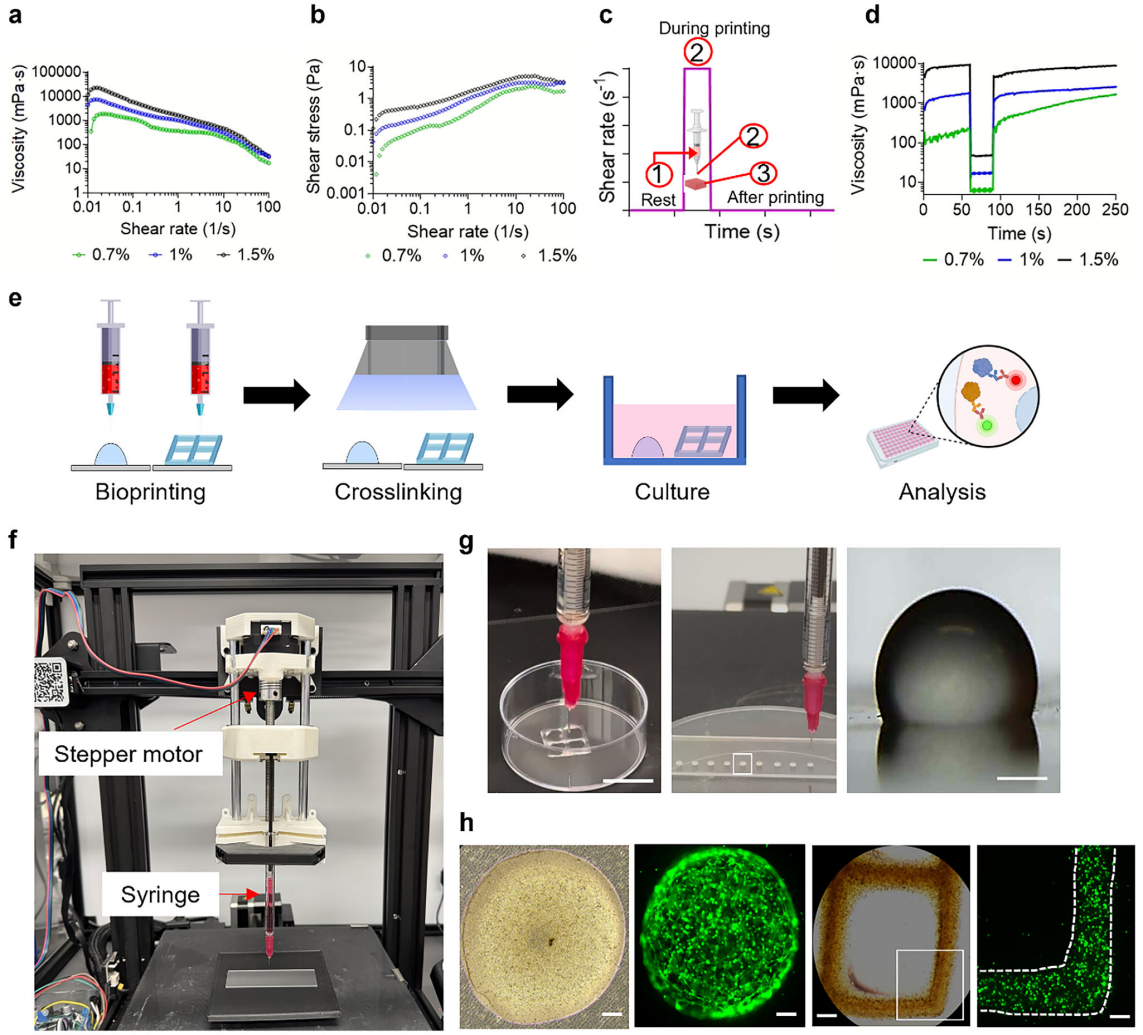
a) Shear‐dependent viscosity curves of KdMA hydrogels with varying polymer concentrations, obtained through dynamic rheological measurements. b) Flow curves showing shear stress versus shear rate for KdMA hydrogels with different concentrations. c) Schematic representation of the testing conditions used in the three‐interval thixotropy test. d) Three‐interval thixotropy test results demonstrating shear‐thinning behavior and structural recovery of bioinks with different KdMA concentrations. e) Overview of the extrusion bioprinting process, from printing to post‐printing analysis. f) Developed piston‐driven extrusion bioprinter with system modifications for enhanced performance. g) Representative images of bioprinted lattice structures and cell‐laden droplets, Scale bar = 1 cm (lattice); 1 mm (droplets). h) From left to right: 1) Bright‐field image showing cell distribution immediately after printing, 2) fluorescence image after one week of culture showing homogeneous cell elongation within the droplets, 3) magnified view of the bottom‐right region of the bioprinted 2 × 2 lattice, and 4) cell viability assessment following printing, Scale bars = 100 µm.

In DLP‐SLA bioprinting, the successful application of kidney dECM‐based bioinks depends on materials with precisely optimized rheological properties.^[^ ^47^ ^]^ To ensure optimal printability and structural fidelity, viscosity and crosslinking kinetics were assessed through rheological analysis. **Figure** **6**a demonstrates the shear‐thinning behavior of the developed bioink. Across all tested concentrations, the bioink exhibited a decreasing viscosity trend with increasing shear rate, a property that is particularly critical for extrusion‐based bioprinting. Under high shear conditions, such as during extrusion through a nozzle, the reduction in viscosity facilitates smooth deposition, while the subsequent recovery of viscosity ensures structural integrity post‐printing. Figure 6b illustrates the relationship between shear stress and shear rate, essential for fluid model analysis. The slope of this graph corresponds to viscosity, and as the shear rate increases, the curve progressively flattens, further confirming the shear‐thinning behavior of the bioink. Additionally, flow does not initiate at zero shear stress but only above a certain threshold (τ_y_), indicating that the bioink possesses yield stress. This characteristic is crucial for maintaining structural stability after printing, as the presence of yield stress ensures that the bioink remains stationary below a specific stress threshold, thereby enhancing shape fidelity.

As shown in Figure S2 (Supporting Information), bioinks with higher KdMA concentrations exhibited increased viscosity, which can be attributed to the higher polymer density and enhanced intermolecular interactions, such as hydrogen bonding and physical entanglements between polymer chains. The increased availability of methacrylate functional groups may also contribute to a more interconnected network, further influencing viscosity. According to the literature, the viscosity of resins optimal for SLA bioprinting should not exceed 5000 cP.^[^ ^48^ ^]^ Figure S2 (Supporting Information) shows that the viscosity of 0.7% KdMA was measured at 341.01 mPa.s, 1% KdMA at 1328.10 mPa.s, and 1.5% KdMA at 4494.27 mPa.s. Among these, the viscosity of 1.5% KdMA approaches the upper viscosity limit for SLA bioprinting, suggesting that the 1.5% KdMA bioink formulation may not be ideal for SLA bioprinting applications. However, the pronounced shear‐thinning behavior exhibited by the 1.5% KdMA sample suggests its potential in extrusion‐based 3D bioprinting using dECM‐based bioink.

To mimic bioprinting conditions, viscosity was measured as a function of time under three distinct phases: at rest, during printing, and after printing (Figure 6c). Before extrusion, the bioink remains in a static state within the syringe, maintaining high viscosity to prevent unwanted flow or leakage from the nozzle. Upon the application of shear stress (e.g., during nozzle extrusion), the bioink exhibits shear‐thinning behavior, reducing viscosity to facilitate smooth deposition. This property is crucial for preventing nozzle clogging while ensuring uniform bioink flow. Finally, after deposition, the bioink undergoes viscosity recovery due to its thixotropic nature. The speed and extent of viscosity recovery determine the stability of the printed structure. Rapid viscosity recovery is essential to prevent spreading or deformation after printing, ensuring high resolution and structural fidelity. The relationship between shear rate and time was analyzed to further evaluate the thixotropic nature of the bioink (Figure 6d).

The observed shear‐thinning and thixotropic properties are key rheological characteristics for bioinks used in extrusion‐based bioprinting.^[^ ^49^ ^]^ Shear‐thinning behavior reduces viscosity under shear stress, allowing smooth extrusion through the nozzle, while viscosity recovers after deposition prevents sagging and deformation. These properties are particularly critical for achieving high shape fidelity in complex 3D‐printed structures. Similar trends have been reported in other ECM‐derived bioinks, where shear‐thinning behavior enables uniform bioink flow, and rapid viscosity recovery supports precise layer stacking.^[^ ^50^ ^]^ However, an excessively rapid recovery rate could hinder proper bioink fusion between printed layers, potentially affecting construct integrity. Future optimization efforts may involve fine‐tuning the bioink's thixotropic recovery time to balance structural fidelity and interlayer adhesion.

Additionally, thixotropic recovery is highly dependent on polymer concentration, molecular interactions, and crosslinking density.^[^ ^51^ ^]^ Increased methacrylation and polymer entanglement typically result in faster recovery, contributing to enhanced structural stability. However, this also increases the risk of higher extrusion resistance, which may require adjustments in printing parameters such as nozzle size or extrusion pressure. Further investigations will focus on quantifying the long‐term stability of printed constructs, particularly in multi‐layered structures, to assess how thixotropic recovery influences the final architecture of bioprinted tissues. This will be essential for optimizing bioink formulations for applications in renal tissue engineering and organoid maturation.

### 2.9 Extrusion‐Based Bioprinting

To evaluate the printability of the developed bioink in extrusion‐based bioprinting, a systematic workflow was established. This process involved bioink deposition, crosslinking, cell culture, and post‐analysis to assess structural integrity and biocompatibility. Figure 6e provides an overview of the extrusion‐based bioprinting process conducted in this study. The process begins with the extrusion of bioink into predefined structures, including droplet and 2×2 lattice patterns, followed by photocross‐linking using a 405 nm light source. The crosslinked, cell‐encapsulated scaffolds are then cultured in a cell culture dish for a designated period to assess cellular behavior and viability. Subsequent post‐analysis, including staining and histological evaluation, is performed to characterize the printed scaffolds.

An in‐house modified piston‐driven extrusion‐based bioprinting system was developed for this study. As shown in Figure 6f, a piston‐driven extrusion 3D bioprinter was adapted for this study. This setup enabled more precise deposition of bioinks in the form of cellular droplets, such as spheroids and organoids. Compared to the in‐house modified pneumatic‐based extrusion bioprinter, it allowed for the fabrication of smaller and more defined structures.^[^ ^52^ ^]^ To validate the printability of the developed bioink, the modified bioprinter was used to load a syringe filled with KdMA bioink. The bioink was then bioprinted into predefined structures, including a 2 × 2 lattice and droplet pattern, confirming its extrusion‐based printability (Figure 6g and Movie S3, Supporting Information). As shown in Figure 6h, the first image (from the left) shows cells encapsulated within the bioprinted droplets immediately after printing. The second image, captured using fluorescence microscopy after one week of culture, reveals that the cells elongated, indicating active spreading and adaptation within the printed environment. The third and fourth images illustrate cell proliferation and growth within the mesh‐patterned bioprinted scaffold, further demonstrating the bioink's versatility and biocompatibility.

The successful extrusion and maintenance of structural integrity in the bioprinted droplet and lattice structures confirm the printability of KdMA bioink. Achieving precise filament formation and droplet deposition is critical for extrusion‐based bioprinting, as it directly influences construct resolution, cell distribution, and overall bioprinted scaffold functionality. The shear‐thinning behavior of the bioink plays a key role in extrusion‐based printing, ensuring smooth flow through the nozzle during deposition while allowing for rapid viscosity recovery post‐printing. This is essential for preventing unintended spreading, enabling high shape fidelity, and ensuring that the scaffold retains its predefined architecture. Additionally, the presence of methacrylate functional groups facilitates rapid photocross‐linking, stabilizing the structure immediately after deposition.

The biocompatibility of the printed scaffolds is evident from the fluorescence microscopy images, where cell elongation and proliferation were observed over the culture period. Elongation of encapsulated cells suggests that the bioink provides a permissive microenvironment for cellular attachment and spreading, while the observed proliferation in the mesh‐patterned scaffold indicates that the bioink can support sustained cell growth. This is particularly important for tissue engineering applications, where the bioink must not only maintain printability but also provide a supportive environment for encapsulated cells.

In previous studies on ECM‐based bioinks, highly substituted methacrylated bioinks often faced challenges related to excessive stiffness and limited cellular activity due to dense crosslinking,^[^ ^53^, ^54^ ^]^ However, in this study, the balance between mechanical integrity and bioactivity was successfully maintained, as evidenced by both high print fidelity and active cell behavior post‐printing. The versatility of the KdMA bioink allows its potential application in fabricating diverse renal tissue models, including kidney organoids and nephron structures. However, further optimization of printing parameters such as nozzle diameter, extrusion pressure, and crosslinking conditions could enhance its application across various bioprinting techniques beyond extrusion, such as SLA‐based bioprinting.

## 3 Conclusion

This study successfully demonstrates the development and application of a stiffness‐tunable, photocross‐linkable, kidney‐specific ECM‐based bioink for kidney tissue bioprinting. The decellularization process effectively removed all cellular components while preserving the structural and biochemical integrity of the kidney ECM, ensuring an organ‐specific microenvironment conducive to cellular growth and maturation. The resulting dECM‐based bioink exhibited favorable rheological properties, making it well‐suited for both DLP‐SLA and piston‐driven extrusion bioprinting techniques.

Furthermore, HEK cells encapsulated within the bioink maintained high viability and proliferative capacity, demonstrating its potential to support long‐term tissue‐specific maturation. Notably, the kidney‐specific ECM provided by this bioink actively promoted HEK cell organization, maturation, and tissue formation, reinforcing its suitability for engineering functional kidney tissue constructs. The bioprinted kidney scaffolds exhibited high structural fidelity, cellular proliferation, and functional tissue development, further validating the bioink's efficacy in renal tissue engineering applications.

Looking ahead, 3D bioprinting strategies utilizing kidney dECM‐based bioinks hold significant promise for advancing bioengineered kidney tissue constructs, with potential applications in regenerative medicine, precision medicine, and disease modeling. This approach could enable therapeutic applications such as drug testing and nephrotoxicity screening while also serving as a foundation for developing bioengineered alternatives to kidney transplantation. Ongoing research aims to optimize the functionality of bioprinted renal tissues by integrating iPSC‐derived kidney organoids with dECM‐based bioinks. To enable more rigorous biological and functional evaluations, future studies will incorporate more physiologically relevant cell types, such as primary kidney cells, proximal tubule epithelial cells, or human iPSC‐derived kidney cells. Specifically, we will conduct comprehensive biological validation, including gene expression profiling to assess lineage‐specific differentiation, immunohistochemical staining for renal‐specific markers such as E‐cadherin, aquaporin‐1, and nephrin, and functional assays such as albumin uptake and barrier integrity, to confirm the physiological relevance of the constructs for future therapeutic applications.

## 4 Experimental Section

### Preparation of Kidney dECM Bioink

The kidney dECM was developed through modifications and further optimization of previously reported protocols,^[^ ^17^, ^23^ ^]^ Fresh porcine kidney tissue was purchased on the day of slaughter from a local butcher, transported on ice to the laboratory, and processed by removing the renal capsule and perirenal fat before freezing. The kidney tissue was then stored at −20 °C to facilitate consistent slicing. The following day, the tissue was sliced into 0.1–0.3 cm thick sections using a meat slicer. To remove blood, the sliced kidney tissue was washed three times for 30 min each with distilled water at a volume ten times greater than the tissue volume in a 3500 mL beaker. The tissue was then treated with 0.5% Triton X‐100 (VWR, Mississauga, ON, Canada) in 1 M NaCl (Sigma–Aldrich, St. Louis, MO, USA) for 16 h. The next day, the tissue was rinsed three times for 1 hour each with distilled water.

Following this, the tissue was stirred in a DNase I (MilliporeSigma, Oakville, ON, Canada) solution at 37 °C for 6–7 h, and then washed with PBS (MilliporeSigma, Oakville, ON, Canada) for an additional 12 h. DNase was an important enzymatic agents that cleave nucleic acid sequences, facilitating the removal of nucleotides after cell lysis in tissues. On a subsequent day, the tissue was treated with a 0.1% peracetic acid solution (MilliporeSigma, Oakville, ON, Canada) for 1 h under agitation, followed by three washes with distilled water, each lasting 30 min. At this stage, the kidney tissue, initially pink, had turned transparent. The decellularized kidney tissue was then frozen at −20 °C for one day and freeze‐dried for three days. The kidney dECM, finely ground to achieve an ultra‐small particle size, was dissolved at a concentration of 1 mg mL^−1^ in 0.5 M acetic acid containing pepsin, stirred at 1000 revolutions per minute (RPM) at room temperature, and mixed for 48 h.

As precipitation occurred immediately upon NaCl addition, the mixture was manually stirred with a spatula for 5 min to ensure thorough mixing, as magnetic stirring was insufficient. The entire solution was distributed and then centrifuged at 10 000 RPM for 15 min, after which the precipitated tissue was collected. The collected precipitated mass was transferred into a 3.5 kDa dialysis tube and dialyzed at 4 °C with continuous stirring at 500 RPM. The dialysis process was performed by replacing the distilled water twice daily, in the morning and evening, to effectively remove residual acetic acid. Following dialysis, the sample was freeze‐dried to ensure a complete solubilization step. A detailed description of the next step, the methacrylation process, was provided in Section 2.3.

### Methacrylate Synthesis

The methacrylation process for the solubilized dECM was conducted as follows: 1 g of kidney dECM was dissolved in 100 mL of 0.5 M acetic acid at room temperature. To optimize methacrylation activation, 5 M NaOH was added to adjust the pH of the solution to 8–9. Methacrylic anhydride (Sigma–Aldrich, St. Louis, MO, USA) was then introduced at a concentration of 2.5 mL per gram of dECM, added dropwise to ensure controlled reaction conditions. The solution was stirred continuously at room temperature for 2 days to facilitate synthesis. Following this, the reaction product was subjected to dialysis using a 3.5 kDa dialysis tube (Thermo Fisher Scientific, Waltham, MA, USA) at 4 °C for three days, with water replaced twice daily. The final sample was freeze‐dried for three days to obtain the final KdMA.

The DS of the synthesized KdMA was quantified using proton nuclear magnetic resonance (^1^H NMR) spectroscopy, ensuring the efficiency and consistency of the methacrylation process. The kidney dECM and KdMA solution was prepared at a concentration of 0.5% (w/v) 1 mL of deuterium oxide (MilliporeSigma, Oakville, ON, Canada). Next, ^1^H NMR spectra for the synthesized KdMA samples were recorded using a 600 MHz NMR spectrometer. The internal reference was set to hydroxyl signals (0.5–1 ppm). The peaks corresponding to primary amine groups (5–6 ppm) were integrated to calculate the DS.

### KdMA Bioink Preparation

Three different concentrations of KdMA were dissolved in sterilized PBS. To prepare these solutions, a 0.5% (w/v) stock solution of LAP (Sigma–Aldrich, St. Louis, MO, USA), as photo initiator, was first prepared. KdMA was added to the stock solution, which consisted of PBS containing 0.5% (w/v) LAP photoinitiator, at concentrations of 0.7%, 1%, and 1.5% (w/v) and stirred at room temperature for three days to ensure uniform mixing. The LAP photoinitiator consists of lithium ions and an acyl phosphinate group, which combine to be activated by ultraviolet or visible light (particularly at the wavelength of 405 nm) to initiate polymerization. Due to its high photoinitiation efficiency and low toxicity, LAP was frequently used in biomaterials research and applications. The exposure duration was varied for each bioink combination based on its photocross‐linking characteristics.

### Histological Analysis

To visualize and analyze residual porcine cells and microarchitecture in tissues following the decellularization process, both untreated native kidney tissue and kidney dECM were fixed in 4% (wt/vol) paraformaldehyde solution (MilliporeSigma, Oakville, ON, Canada) at room temperature for 24 h. The samples were then dehydrated by sequential immersion in ethanol solutions of 100%, 95%, 90%, 75%, 70%, and 50% (vol/vol) overnight. Subsequently, the tissues were embedded in paraffin. For further staining procedures, the paraffin‐embedded tissue samples were sectioned into thin slices with a thickness of 5–7 µm. These sections were stained with H&E (VWR, Mississauga, ON, Canada) and Masson's trichrome stain (Polysciences Inc., Warrington, PA, USA). Staining enabled the detection of any residual cellular components and provided insight into the preservation of structural integrity in the decellularized tissue. This approach ensured a comprehensive evaluation of the effectiveness of the decellularization process and the structural characteristics of the tissue. The samples of the H&E and Masson's trichrome were performed and imaged using an inverted microscope (ECHO Revolve, San Diego, CA, USA). This process enabled the visualization of nuclei within the kidney tissue and was used to evaluate the presence or absence of nuclei in the dECM samples.

### Biochemical Analysis

The quantification of DNA and TPC was performed using the PicoGreen dsDNA Assay Kit (ThermoFisher, Waltham, MA, USA) and the TPC kit (ThermoFisher, Waltham, MA, USA), respectively, to evaluate the residual cellular and protein content in dECM (*n* = 3). For this analysis, 50 mg of native kidney tissue and lyophilized kidney dECM were added to 1 mL of a pepsin digest solution (0.1 mg mL^−1^, Ward's Science, ON, Canada). The mixture was homogenized using an FSH‐2A High‐Speed Homogenizer at 20000 RPM. Subsequently, the samples were vortexed and incubated in a 65 °C water bath for 6 h. After incubation, the samples were centrifuged at 4000 RPM for 15 min, and the supernatant was collected. Measurements were performed using a microplate reader (SpectraMax M3, Molecular Devices, San Jose, CA, USA) according to the manufacturer's protocol.

### Physical Property Characterization

The mechanical properties of the hydrogel were evaluated through a comparative analysis of the compressive modulus of crosslinked hydrogels. Compression testing was conducted using a flat cylinder probe with a diameter of 12.5 mm. To prepare the samples, 2 mL of hydrogel precursor solution with varying KdMA concentrations was transferred into cylindrical molds with a diameter of 8 mm and a height of 4 mm (Figure S4a, Supporting Information). Each well received 400 µL of prepolymer solution, which was cross‐linked for 30 s in the DLP‐SLA bioprinting system, to ensure fully crosslinking of the cylindrical samples (Figure S4b, Supporting Information). Compression was applied to the hydrogel surface up to 80% strain, and force‐displacement data were recorded. The compressive modulus was determined using the initial dimensions of the hydrogel samples and a custom MATLAB script, with the slope of the linear region corresponding to an initial strain of 10%.

The swelling ratio of the crosslinked hydrogel samples was measured to evaluate their water uptake capability. A total of 400 µL of hydrogel solutions with different concentrations were cast into molds with a diameter of 8 mm and a height of 4 mm and cross‐linked using a DLP‐SLA bioprinter for 30 s. The cross‐linked hydrogels were then immersed in PBS and incubated at 37 °C. At predetermined time points, the samples were removed and the hydrated weight (W_w_) of each sample was recorded. Subsequently, the samples were frozen at −20 °C and lyophilized for three days to obtain the dry weight (W_d_). The swelling ratio was then calculated using the following equation:

(1) $$Swelling\;ratio=\frac{W_{w}-W_{d}}{W_{d}}\times 100$$

To investigate the degradation behavior of the samples, hydrogels were prepared using the previously described method,^[^ ^21^, ^55^ ^]^ All samples were freeze‐dried for three days, and their initial dry weight (W_o_) was recorded. The samples were then immersed in PBS at 37 °C. At predetermined time points, samples were retrieved for degradation analysis. The retrieved samples were frozen at −20 °C and subsequently freeze‐dried for three days. The weight of the freeze‐dried samples was then measured to determine the remaining polymer matrix weight after degradation (W_r_). The degradation ratio was expressed as the percentage of the remaining weight of the sample after degradation. The equation used for the calculation is as follows:

(2) $$Degradation\;ratio=\frac{W_{r}}{W_{0}}\times 100$$

### Microstructure Analysis

The microstructure of the hydrogels was meticulously examined using SEM. Hydrogel samples were prepared according to previously mentioned process, followed by freezing at −20 °C and lyophilization for three days. To capture naturally fractured cross‐sections, the freeze‐dried samples were partially sectioned with a sharp blade, allowing the remaining portions to fracture spontaneously. The fractured surfaces were positioned with the exposed surfaces facing upwards and carefully mounted onto SEM stubs to ensure proper alignment for imaging. Before imaging, the samples were sputter‐coated with a 15 nm thin layer of gold to enhance conductivity and minimize charging effects. High‐resolution imaging was then performed using a Phenom Pro X SEM, allowing for detailed visualization and analysis of the microstructural characteristics of the hydrogels. Pore size analysis was conducted using ImageJ software. The image scale was calibrated using the straight‐line tool based on the SEM scale bar. To ensure clear separation between pores and background, the images were preprocessed and converted to binary format, followed by noise removal. The analysis particles function was then used to quantify pore features such as area, circularity, and centroid coordinates. For each identified pore, two perpendicular diameters were measured, and their average was used to represent the pore size.

### Characterization of Cross‐linking Kinetics and Rheological Properties

The rheological properties of KdMA at varying concentrations were meticulously characterized using a rheometer (MCR 302, Anton Paar, Graz, Austria). All measurements were performed at ambient temperature employing a parallel plate geometry with a 25 mm diameter and a 0.5 mm gap maintained on the rheometer platform. To evaluate photocross‐linking kinetics, a 405 nm light source was applied from beneath to irradiate a thin prepolymer layer, initiating the crosslinking process. The light exposure began 60 s after the experiment commenced and was maintained for a duration of 5 min. Oscillatory measurements were conducted at a constant 0.5% shear strain and a frequency of 1 Hz, allowing for the simultaneous recording of both storage and loss moduli. After the light exposure phase, frequency sweeps were performed within the range of 0.1–100 rad s^−1^ while maintaining a constant shear strain of 0.5%. This was done to observe the dynamic rheological properties of the bioink as it underwent crosslinking.

To validate the shear‐thinning behavior of the bioink, viscosity measurements were performed by varying the shear rate from 0.01 to 1000 s⁻¹ in the absence of light exposure. Additionally, the three‐interval thixotropy test was employed to assess the thixotropic behavior and phase transitions following exposure to excessive stress. During the first (0–60 s) and third (91–270 s) intervals, low shear stress (0.1 Pa) was applied to stabilize the sample and allow for structural recovery. Conversely, high shear stress was applied during the second interval (61–90 s) to simulate the flow through the nozzle and the structural deformations that occur during extrusion bioprinting.

### Cell Culture

HEK (HEK‐293; CRL‐1573, ATCC, Manassas, VA, USA) cells were maintained in high‐glucose Dulbecco's Modified Eagle Medium (Corning, NY, USA) supplemented with 10% fetal bovine serum (Corning, NY, USA) and 1% penicillin/streptomycin (Cytiva, Marlborough, MA, USA). The cells were cultured in tissue culture flasks and passed as necessary, with growth conditions sustained in a 37 °C incubator with 5% CO_2_. The culture medium was refreshed every two to three days to support optimal cell viability and proliferation.

### Cell Viability

To fabricate cell‐encapsulated scaffolds, cells were seeded at a density of 1 × 10^6^ cells mL^−1^ within various bioinks. Following cross‐linking, the scaffolds were cultured in well plates, with medium changes performed every three days. Cell viability was assessed at the desired time point post‐bioprinting using the LIVE/DEAD Viability/Cytotoxicity Kit (Biotium, Fremont, CA, USA). To ensure thorough removal of residual culture medium, the 3D scaffolds were washed three times with PBS, followed by a 15 min incubation in PBS at room temperature. This additional incubation step was essential as residual medium often remains trapped within the porous structure of 3D scaffolds despite washing, bringing background signals in the images. Extended exposure to PBS facilitates the diffusion and removal of any remaining medium, ensuring more consistent and uniform staining.

For viability assessment, the cell‐laden scaffolds were stained using a solution of 0.5 µL mL^−1^ calcein AM and 2 µL mL^−1^ ethidium homodimer (EthD) in 1 mL PBS. The staining solution was incubated in the dark at room temperature for 30 min. After staining, scaffolds were washed three times with PBS to eliminate any unbound reagents. Fluorescence imaging was then performed using an inverted fluorescence microscope to visualize live (calcein AM) and dead (EthD) cells, as well as the overall distribution of cells within the scaffolds. Calcein AM fluorescence was detected using the FITC channel, while EthD fluorescence was visualized using the TxRed channel. High‐resolution 3D imaging and z‐stack reconstruction of the cell‐encapsulated hydrogels were carried out using a Nikon Eclipse Ti confocal microscope (Nikon, Tokyo, Japan).

### Biocompatibility Analysis

To evaluate cell proliferation within scaffolds fabricated from the KdMA bioink, HEK cells were encapsulated and cultured for one month. The bioink, seeded at a density of 1 × 10^6^ cells mL^−1^, was loaded into a syringe mounted on an extrusion‐based bioprinter, and an appropriate volume of bioink was dispensed. Multiple samples with a diameter of 2 cm and a thickness of 200 µm were printed to ensure that at least one dimension fell within a range comparable to the critical limit for medium diffusion. Immediately after printing, crosslinking was performed using 405 nm light. The crosslinked discs were transferred to a petri dish and thoroughly washed multiple times with PBS to remove any uncross‐linked bioink. Finally, the samples were transferred to an incubator and cultured for one month, with the culture medium replaced with fresh medium every three days.

To evaluate cell morphology and proliferation, at least three smaller discs, approximately 4 mm in diameter, were created from each sample using a disc punch. The samples were stained for cytoskeleton and nuclear visualization at 5, 10, and 30 days of culture using Phalloidin (Cytoskeleton Inc., Denver, CO, USA) and DAPI (MilliporeSigma, Oakville, ON, Canada), respectively. Before staining, the samples were fixed in 4% v/v paraformaldehyde for 90 min. Permeabilization was achieved using 0.5% v/v Triton X‐100 for 20 min to enhance membrane permeability, facilitating the penetration of staining reagents into the cells. The samples were then stained in the dark at room temperature for 90 min with Phalloidin 488. Following staining, the samples were mounted using a mounting medium containing DAPI and imaged using a fluorescence microscope equipped with DAPI and FITC channels.

### Proliferation Assay

To evaluate cellular proliferation and assess mitochondrial metabolic activity, the tetrazolium hydroxide salt assay (XTT kit, Biotium, Fremont, CA, USA) was employed using the manufacturer's protocol. Briefly, the XTT reagent was added to the culture medium at a 1:10 ratio to enable the measurement of cellular viability and metabolic activity. The samples were incubated with the XTT solution for 24 h at 37 °C in a 5% CO_2_ atmosphere to allow for sufficient reduction of the tetrazolium salt by metabolically active cells. Following incubation, 100 µL of the supernatant from each well was carefully transferred to a 96‐well plate to minimize potential interference from cellular debris. The metabolic activity of the cells was then quantified by measuring the absorbance of the reduced formazan product, which forms as a result of the mitochondrial enzymatic reduction of the XTT reagent. Absorbance readings were taken at a wavelength of 450 nm using a spectral scanning plate reader, with the absorbance intensity correlating with the number of viable cells and their metabolic activity. This method provides a reliable and quantitative assessment of cellular proliferation and mitochondrial function in response to biomaterial or experimental conditions.

### DLP‐SLA Bioprinting

A DLP‐SLA bioprinting system was utilized to fabricate multilayered scaffolds. A 405 nm light beam projector (PRO4500, San Marcos, CA, USA) was mounted on a custom‐developed light stand, enabling precise exposure control during the bioprinting process. The beam projector was connected to a laptop containing the printing design, which controlled the light pattern for photopolymerization. The bioink‐loaded plate was positioned on a Z‐axis stage, facilitating layer‐by‐layer bioprinting, with the stage moving upward by 10 µm after each layer was printed. Each bioprinted scaffold was fabricated with a single‐layer volume of 100 µL, and a total of five layers were sequentially printed to achieve the desired structure. The controlled light exposure and stage movement enabled high‐resolution printing of multilayered scaffolds with uniform thickness.

### Extrusion Bioprinting

A piston‐driven extrusion bioprinter was custom‐developed, utilizing a stepper motor as the primary actuator, with surrounding components fabricated using 3D printing to create a protective casing (Movie S4, Supporting Information). Bioprinting was performed using a 500 µL Hamilton syringe coupled with a 25 G nozzle. For droplet‐based bioprinting, the droplet volume was precisely controlled by optimizing the rotation cycles of the stepper motor. In contrast, for 2 × 2 lattice‐pattern bioprinting, the stepper motor continuously rotated from the start to the end of the G‐code execution, enabling the continuous dispensing of the bioink during scaffold fabrication. Since a photocross‐linkable bioink was used, printed structures were cross‐linked using a 405 nm light source to ensure structural stability.

### Statistical Analysis

Quantitative data are reported as mean ± standard deviation and inferential statistics (p‐values) were used for further analysis. Statistical significance was determined using one‐way ANOVA, with significance levels set at p < 0.05, p < 0.01, or p < 0.001. To identify specific differences between group means, Tukey's HSD test was used for post hoc analysis. The analysis was performed using GraphPad Prism version 12.2.2 software.

## Conflict of Interest

The authors declare no conflict of interest.

## Author Contributions

J.S. designed and performed the experiments, analyzed the data, and drafted and finalized the manuscript. N.T.R. assisted with the experiments, contributed to data analysis and interpretation, and helped edit the manuscript. S.C. and Z.L. provided assistance with the experiments, and Z.L. also contributed to manuscript editing. D.‐H.K. contributed to manuscript editing and provided critical review comments. K.K. conceptualized and supervised the study, secured funding, and reviewed and edited the manuscript. All authors reviewed and approved the final version of the manuscript.

## Supporting information



Supporting Information

Supporting Information

Supporting Information

Supporting Information

## Data Availability

The data that support the findings of this study are available from the corresponding author upon reasonable request.
