# Supplementary material for: Photocrosslinkable Kidney Decellularized Extracellular Matrix‐Based Bioink for 3D Bioprinting
Source: Adv Healthc Mater. 2025 Jul 16;14(24):2501616. doi: 10.1002/adhm.202501616 (PMC12447028; doi:10.1002/adhm.202501616)
Supplement: Supplementary file 1 — Supporting Information [file ADHM-14-0-s002.docx]

Supplementary Materials for

Photocrosslinkable Kidney Decellularized Extracellular Matrix-based Bioink for 3D Bioprinting

Jaemyung Shin^1^, Nima Tabatabaei Rezaei^2^, Subin Choi^1^, Zhangkang Li^3^, Deok-Ho Kim^4^, and Keekyoung Kim^1,2 *^

^1^Department of Biomedical Engineering, Schulich School of Engineering, University of Calgary, Calgary, Alberta T2N 1N4, Canada

^2^Department of Mechanical and Manufacturing Engineering, Schulich School of Engineering, University of Calgary, Calgary, Alberta T2N 1N4, Canada

^3^Basic Medical Research Center, Medical School of Nantong University, Co-Innovation Center of Neuroregeneration, Nantong 226001, Jiangsu Province, China

^4^Department of Biomedical Engineering, Johns Hopkins University, Baltimore, Maryland, USA

**^*^Corresponding author:** Dr. Keekyoung Kim, email: [keekyoung.kim@ucalgary.ca](mailto:keekyoung.kim@ucalgary.ca)


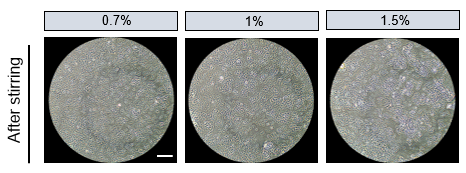


**Figure S1.** Microscopic image showing the homogeneous fiber state of the developed bioink after mixing for the optimized stirring period. Scale bar = 200 μm


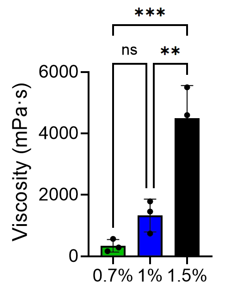


**Figure S2.** Viscosity measurement of KdMA at varying concentrations. Error bars represent standard deviation (*n*=5).


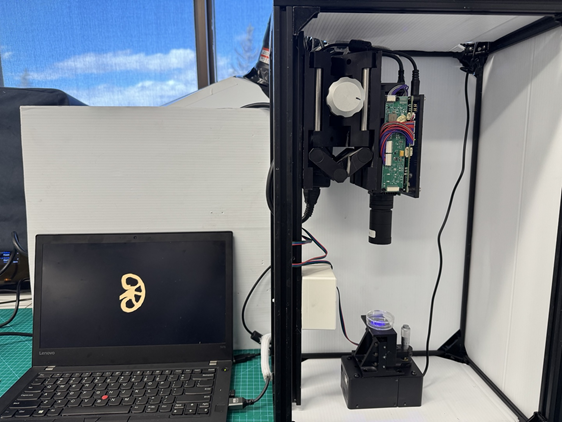


**Figure S3.** Digital light processing-based stereolithography bioprinting system connected to a computer for design file implementation.


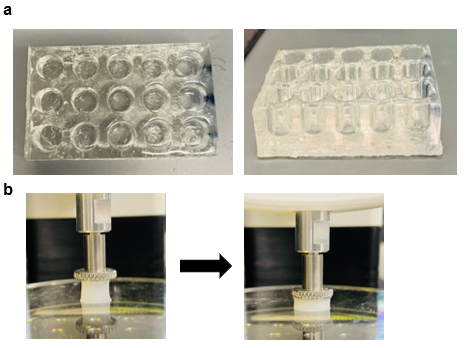


**Figure S4.** (a) Polydimethylsiloxane (PDMS) mold designed for the fabrication of cylindrical scaffolds used in mechanical property evaluation. The left panel displays a top view, while the right panel shows a side view. (b) Mechanical testing of a crosslinked cylindrical hydrogel sample to assess compressive strength.
